# Beyond the Scalpel: the Role of Image-Guided Thermal Ablation in Management of Metastatic Renal Cell Carcinoma in the Era of Immunotherapy

**DOI:** 10.1007/s00270-025-04101-y

**Published:** 2025-06-24

**Authors:** Kin Fen Kevin Fung, Philippe Barthelemy, Fabien Moinard-Butot, Mathilde Airoldi, Roberto Luigi Cazzato, Afshin Gangi

**Affiliations:** 1 Department of Radiology, Hong Kong Children’s Hospital, Kowloon, Hong Kong; 2 Department of Medical Oncology, University Hospital of Strasbourg, 1, Place de L’hopital, 67000 Strasbourg, France; 3 Department of Medical Oncology, Institut de Cancérologie Strasbourg Europe, Strasbourg, France; 4 Department of Interventional Radiology, University Hospital of Strasbourg, 1, Place de L’hopital, 67000 Strasbourg, France; 5 School of Biomedical Engineering and Imaging Sciences, King’s College London, London, UK

**Keywords:** Renal cell carcinoma, Immunotherapy, Thermal Ablation, Cryoablation, Metastasis

## Abstract

Renal cell carcinoma (RCC) is the most common type of kidney cancer and accounts for approximately 90% of all renal malignancies. About 30% of patients have metastatic disease at their initial presentation. Historically, these patients have very poor prognosis with a median survival of 12 months. The recent introduction of immune checkpoint inhibitors (ICI)-based immunotherapy, which disrupts cancer-induced immune tolerance and promotes immune-mediated cancer cell killing, has significantly improved patient outcome. While ICI-based therapy represents the standard of care for metastatic RCC, there are significant treatment-related adverse effects. This review article will examine how image-guided ablation, as an adjunct to immunotherapy, can improve survival and quality of life in patients with metastatic RCC.

## Background

Renal cell carcinoma (RCC) is the most common type of kidney cancer and accounts for approximately 90% of all renal malignancies [1]. During the past two decades, there has been an annual increase of 2% in RCC incidence both worldwide and in Europe, with approximately 99,200 new RCC cases and 39,100 kidney cancer-related deaths in the EU in 2018 [1, 2]. Three main types of RCC exist, the most common one being clear cell RCC (70 – 80%), followed by non-clear cell carcinoma including papillary RCC (10 – 15%) and chromophobe RCC (4 – 5%), with variable cancer-specific survival and prognosis. Most patients are diagnosed with localised disease amenable to surgical treatment with definitive intent. However, approximately one-third of patients treated with curative intent will develop metastatic disease. Likewise, 30% of patients have metastatic disease at their initial presentation [3]. According to the International Metastatic Renal Cell Carcinoma Database Consortium (IMDC), the most common metastatic sites (in descending order) include lungs (71%), lymph nodes (49%), bones (36%), liver (21%), adrenal (9%), brain (9%), pancreas (5%), pleura (4%) and thyroid (0.6%) [4].

Historically, metastatic clear cell RCC had very poor prognosis with a median survival of 12 months [5]. The IMDC score was developed as a prognostic tool in advanced RCC. Using six clinical and laboratory risk factors, the IMDC scoring system classifies patients into three risk categories: favourable, intermediate and poor, which can be used to estimate prognosis and guide treatment decisions in first-line systemic therapy [6]. In the past two decades, the introduction of novel systemic options has transformed the treatment landscape of metastatic RCC (mRCC) with significant improvement in patient survival. These include immune checkpoint inhibitors (ICI), anti-vascular endothelial growth factor (anti-VEGF) tyrosine kinase inhibitors (TKI) mammalian target of rapamycin inhibitors (mTOR-I) and recently hypoxia-inducible factor 2 alpha (HIF2α) inhibitors [7, 8]. Anti-programmed cell death protein 1 (PD-1)-based combination therapy with TKI or anti-T-lymphocyte-associated protein 4 (CTLA4) agents is considered the standard recommended first-line treatment in patients who require systemic therapy in the American Society of Clinical Oncology (ASCO) and European Society for Medical Oncology (ESMO) guidelines [6, 9]. The biological basis of ICI in treatment of metastatic clear cell RCC is reliant on the abundant tumoural expression of programmed cell death ligand-1 (PD-L1), which is responsible for evading from the host immune mechanisms. The binding of programmed cell death ligand-1 (PD-L1) on RCC tumour cells and programmed cell death-1 (PD-1) on anti-tumour T cells cause downregulation of cytotoxic killing activity and exhaustion of T cells, leading to an immune escape phenomenon. This hypothesis was supported by a meta-analysis of studies examining the association between PD-L1 expression and mortality in RCC patients before the widespread adoption of ICI, the adjusted hazard ratio for cancer-specific death was 1.81 (95% CI 1.31–2.49) for patients with tumoural PD-L1 expression compared with those without [10]. A similar network meta-analysis demonstrated ICI-ICI combination offered the highest likelihood in progression-free survival in patients who demonstrated ≥1% PD-L1 expression [11]. Several large phase 3 open-label multicentre randomised controlled trials of ICI combination (ICI plus ICI or ICI plus TKI) have shown improvement in the overall survival (OS) in the intention-to-treat population, summarised in Table 1 [12–15].

**Table 1:** Key trials evaluating first-line immunotherapy-based treatment for metastatic RCC

Trial name	Phase and design	Treatment arm and control arm	Median progression-free survival (months)	Median overall survival (months)	Hazard ratio for overall survival	Complete response rate	Most common adverse events (grade 3 or above)
CLEAR	Phase 3, open-label, multicentre RCT	Lenvatinib + Pembrolizumab vs. Sunitinib	23.3	33.7	0.72 (95% CI = 0.55 – 0.93)	17.2%	- Hypertension (27.6%) - Diarrhoea (9.7%) - Weight decrease (8.0%)
KEYNOTE-426	Phase 3, open-label, multicentre RCT	Axitinib + pembrolizumab vs. sunitinib	15.7	45.7	0.73 (95% CI = 0.60 – 0.88)	10%	- Hypertension (22.1%) - Diarrhoea (9.1%) - Increase in ALT (13.3%)
CheckMate 214	Phase 3, open-label, multicentre RCT	Nivolumab +_ ipilimumab vs sunitinib	12.4	52.7	0.72 (95% CI = 0.62-0.83)	12%	- Increase lipase (11.7%) - Fatigue (4.4%) - Diarrhoea (3.8%)
CheckMate 9ER	Phase 3, open-label, multicentre RCT	Nivolumab + cabozantinib versus sunitinib	16.6	49.5	0.70 (95% = 0.56 – 0.87)	12.4%	- Hypertension (12.8%) - PPE (7.8%) - Diarrhoea (7.2%)

RCT Randomised controlled trial; PPE palmar–plantar erythrodysaesthesia; ALT Alanine aminotransaminase

The use of these novel systemic therapies is, however, associated with high incidence of treatment-related toxicity due to TKI or/and immune checkpoint inhibitors. The immune-related adverse events are induced by the expansion of activated T cell population infiltrating non-tumoural host organs. Common immune-related adverse effects include diverse events including skin rash, colitis, pneumonitis, hepatitis, endocrine dysfunction and encephalitis. Grade 3 or higher adverse events (according to Common Terminology Criteria for Adverse Events [CTCAE]) have been reported at rates of 55% to 83.1%. Amongst the first-line systemic treatments, the discontinuation rate due to adverse events ranged from 12% to 27.6% [12, 14–16]. Approximately one-third of all patients required high-dose corticosteroid use for management of treatment-related adverse effects [14, 15, 17].

Furthermore, despite the improvement in overall survival rates, overall response rate, ranging from 39.1 to 60%, and complete response (CR), achieved in 10 – 18%, most patients will develop disease progression [11–14, 17, 18]. Resection or ablation of the primary tumour or metastasis has been proposed to improve patient outcomes by achieving a “no evidence of disease” (NED) status or to stop systemic therapies in order to avoid treatment-related toxicities and improve quality of life. A recent retrospective study by Moinard-Butot et al in 80 mRCC patients demonstrated that, by adding such adjunctive local treatment, the CR rate can be improved to 24% by including NED patients. The ESMO guideline suggests that metastasectomy, thermal ablation, stereotactic body radiotherapy (SBRT) and other local treatments can be considered for selected patients with low metastatic burden after multidisciplinary team review [6]. Similar expert consensus opinion was reached in an European Delphi Study with the majority of expert (86%) agreed that treatment of residual disease should be considered whenever it is possible to achieve a state where the patient has no evidence of disease or near-complete response [19]. Given the lack of high-quality evidence comparing locoregional treatment modalities for mRCC, as highlighted by the Tuscany Interdisciplinary Uro-Oncologic Group (GIOTTO), such treatment options should be personalised and discussed in a multidisciplinary setting, considering the patient’s performance status, IMDC score, metastatic burden and tumour histology.

Image-guided thermal ablation is a well-established treatment modality in liver, lung and adrenal metastases in a variety of intra-abdominal cancers [20–22]. In a systematic review including 56 retrospective studies assessing the outcome of surgical metastasectomy in mRCC patients, those who received complete surgical resection of metastases had a longer median OS (36.5 – 142 months) compared those who did not undergo metastasectomy (8.4 – 27 months) [23]. Of note, most of these patients belong to IMDC favourable risk group. Surgical-related morbidity is also not negligible with a major complication (Clavien–Dindo classifications III – IV) rate of 25.1% and an in-hospital mortality rate of 2.4% [24]. While there is lack of trials comparing surgical metastasectomy versus ablation in the context of mRCC, in many other cancer types, ablation has shown to be equally efficacious as surgery with the benefit of shorter hospital stay and possibility of treating patients with multiple comorbidities [25–27]. According to the most recent National Comprehensive Cancer Network (NCCN) Clinical Practice Guidelines in Oncology on mRCC management, thermal ablative therapies can be considered as alternative techniques in patients who are not surgical candidates for metastasectomy [28]. This article aims to the review the different clinical scenarios where image-guided ablation can be used as an adjunct to immunotherapy to improve survival and/or quality of life of patients with mRCC.

## Clinical Scenarios Where Ablation Can be Used as an Adjunct to Immunotherapy

### Synchronous and Metachronous Oligometastases (Figure 1)

**Fig. 1:**
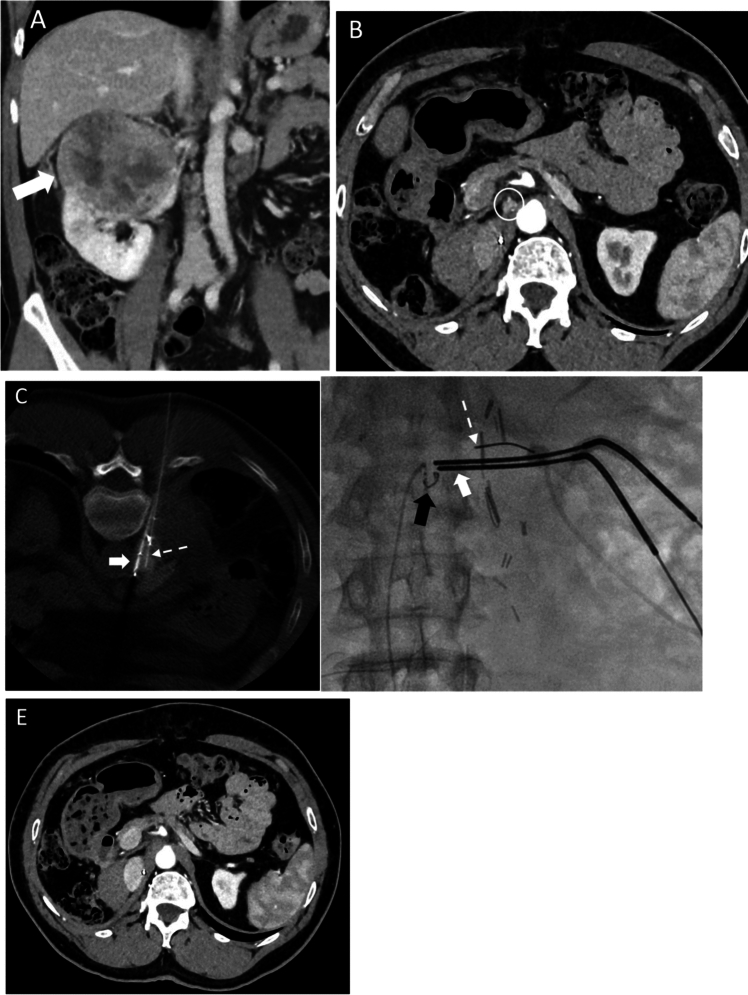
A 67-year-old woman with asynchronous right paraaortic nodal RCC metastasis treated with cryoablation, allowing delay of initiation of immunotherapy. **a** Coronal reformatted contrast-enhanced CT abdomen showed primary right upper pole RCC (white arrow), which was resected by radial nephrectomy. Pathology showed clear cell RCC. **b** Axial contrast-enhanced CT abdomen performed 1 year after right nephrectomy showed right paraaortic nodal metastasis (white circle), **c** and **d** Under (c) CT and (d) fluoroscopic guidance, cryoablation of the right paraaortic nodal metastasis performed using two 17G cryoprobes (white arrow). A 22G spinal needle (dashed white arrow) was used for hydrodissection. Coil embolisation of a parasitic right diaphragmatic artery supplying the node also performed to reduce cold sink effect. **e** axial contrast-enhanced CT at 6 months post-cryoablation confirmed necrosis of nodal metastasis

Oligometastatic RCC was defined as having fewer than 5 lesions of metastatic deposits at time of detection, including both synchronous and asynchronous disease [6, 19]. Synchronous development refers to detection of metastatic deposits at the same time of primary RCC. The definition of asynchronous disease is less well defined but generally considered as detection of metastatic foci at 1 year or more after partial or radical nephrectomy for primary RCC [19, 29].

In IMDC favourable risk patients with indolent metastatic disease, ablation of all metastatic lesions can be considered as an alternative strategy to active surveillance to delay the initiation of ICI-based therapy (Figure 1) [18, 30]. Welch et al described the use of percutaneous image-guided ablation of 82 mRCC lesions in 61 patients, including metastases to the liver, adrenal gland, spine/paraspinal region, bones, nephrectomy bed/retroperitoneum, soft tissue, lung and diaphragm. 80% of lesions were metachronous, and 20% were synchronous. The authors reported 4 technical failure (4.9%) defined by residual tumour or progression observed within 3 months of follow-up. The estimated 3-year OS rate was 76%, and estimated 3-year cancer-specific survival rate was 82%. Only 33 patients (54%) received systemic therapy after ablation. Major complications (i.e. CTCAE grade 3 or 4) were reported to be 4%. After ablation of adrenal metastases, one patient developed deep venous thrombosis and another patient developed splenic bleeding requiring emergent splenectomy [31].

In an earlier study by Bang et al. evaluating the outcome of percutaneous cryoablation for local tumour control in 27 patients in mRCC, similar efficacy was demonstrated with a median OS of 2.69 years and the 3-year OS rate was 45%. Of note, 18 patients (67%) were categorised as IMDC intermediate risk and 9 were of poor risk (33%). None of the patients was considered as surgical candidate for metastasectomy. The major complication rate was reported to be 1.7%, with one patient developing foot drop requiring ankle brace after cryoablation of a metastatic lesion involving the L5 vertebral body [32].

In a pilot randomised trial using a novel immune checkpoint inhibitor tremelimumab (anti-cytotoxic T lymphocyte antigen 4 [CTLA4]) with or without cryoablation in 29 mRCC patients, the addition of cryoablation did not increase treatment toxicity and led to a significant increase immune cell infiltration in the clear cell subtype [33]. Despite the low response rate to tremelimumab, treatment response was shown to be durable in a subset of mRCC with clear cell histology. From an immune monitoring perspective, a subset analysis in patients with clear cell histology demonstrated favourable immunomodulation within the tumour microenvironment when cryoablation was provided immediately prior to the initiation of tremelimumab. There was a significant increase in CD3+ and CD8+ T cells in both tumour and stromal regions of the clear cell mRCC treated with cryo-tremelimumab combination therapy compared with tremelimumab monotherapy alone. The release of intracellular cytokines and other immunogenic signals following necrotic cell death caused by cryoablation can potentially initiate or augment a tumour-specific immune response[33].

In a specific subgroup of patients with advanced RCC and synchronous surgically resectable oligometastatic disease, after nephrectomy and metastasectomy, these patients are rendered M1 with no evidence of disease (M1 NED), who are at high risk of disease recurrence. Adjuvant use of pembrolizumab has demonstrated significantly longer disease-free survival compared to placebo (77.3% vs 68.1%, p = 0.002) and reduced disease recurrence (hazard ratio for recurrence or death, 0.68 [95% confidence interval 0.53 – 0.87]) in this subgroup of patients. In a multidisciplinary tumour board setting, similar considerations could be applied to patients with synchronous oligometastatic deposits who undergo nephrectomy and image-guided ablation of metastases.

### Oligoprogressive Disease

In patients who developed oligometastases despite first-line ICI-based therapy, metastasis-directed ablation may help to improve disease control and avoid the need to switch to second-line or third-line therapy, which can carry significant treatment toxicities. This potential benefit has been demonstrated in the realm of stereotactic body radiation therapy (SBRT), which is also considered as an ablative modality in the NCCN guideline [28]. In a single-arm phase 2 clinical trial of 20 patients on first- to fourth-line systemic therapies with fewer than 3 sites of oligoprogressive disease, patients received upfront and sequential SBRT to a total of 37 sites. Local control was reported in all patients. At a median follow-up of 10.4 months, SBRT extended the duration of ongoing systemic therapy by more than 6 months in 14 patients (70%)[34]. In another multicentre single-arm study, 37 patients with 57 oligoprogressive sites were treated with SBRT following at least 3 months of stable disease on TKI therapy. The median time to change in systemic therapy was 12.6 months [35]. A prospective multicentre randomised trial (GETUG-StORM-01, NCT04299646, ClinicalTrials.gov) is ongoing to evaluate the efficacy of SBRT in prolonging PFS in patients with oligometastatic RCC and in which proportion it can delay the initiation of systemic therapy. A total of 114 patients were expected to be recruited [36]. At this time, there was no published evidence concerning the use of image-guided thermal ablation and further studies are needed to confirm this hypothesis.

### Treatment of Residual Disease (Figure 2)

**Fig. 2:**
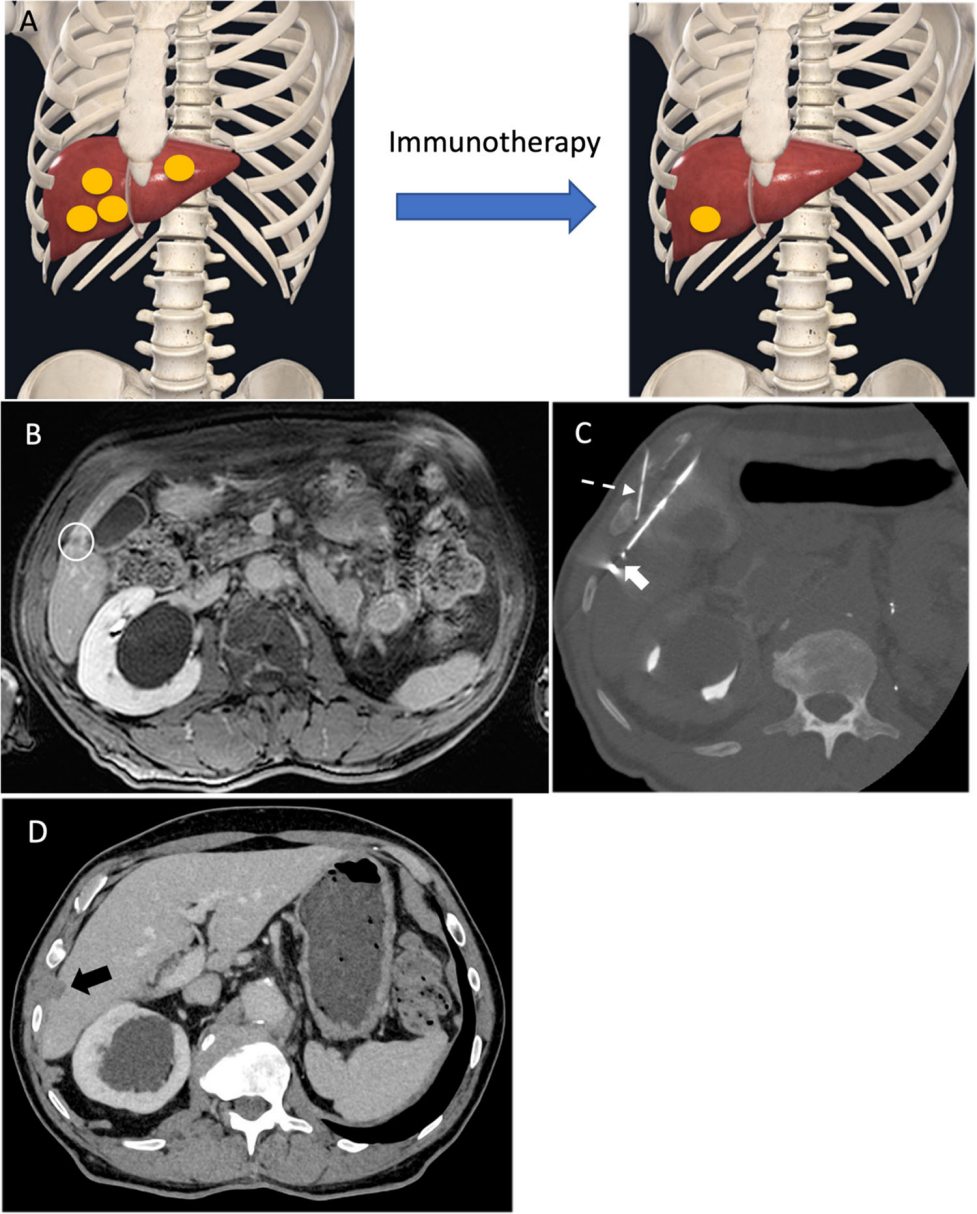
A 70-year-old man who received 18 months of immunotherapy with residual liver metastasis treated with microwave ablation. **a** Graphical illustration demonstrates that, after immunotherapy, the number of liver metastasis reduced from four to one but the patient was unable to reach complete response. **b** Axial T1-weighed post-contrast MR abdomen showed residual metastatic focus at subcapsular region of hepatic segment V (white circle). **c** Under US and CT guidance, the lesion was ablated using 16G microwave antenna (white arrow). A 22G spinal needle (dashed white arrow) was used for hydrodissection. **d** Contrast-enhanced CT performed four years after microwave ablation of liver metastasis confirmed complete disease remission.

In patients with near-complete response of ICI-based combination therapy, deferred nephrectomy and/or resection of residual metastatic sites have been proposed to achieve no evidence of disease (i.e. CR for all lesions). This could potentially lead to the discontinuation of systemic therapy and thereby limit treatment-related toxicity. However, ICI-based combination therapy often results in a severe desmoplastic reaction, which increases perinephric adhesions and thus surgical complexity [18, 37]. Pignot et al reported a promising 2-year PFS and OS rates of 78.3% and 86.1%, respectively, and that 66.7% of patients were free from systemic treatment. However, 10% of patients (3/30) developed major complications, including one surgery-related death due to haemorrhagic shock and hypovolemia with multiorgan failure [38]. Similarly, Moinard-Butot et al reported that, by adding local treatment of residual disease, the CR rate has improved to 24% compared to that of 10% with systemic treatment alone [18]. In this cohort, 60% of residual disease resected was primary tumour on the kidney. Microwave ablation of liver metastasis was reported in one patient (Figure 2).

RFA had been explored as a cytoreductive strategy in patients with metastatic RCC and small primary tumours less than 5cm during the TKI era. The median overall survival (OS) was greater in the cytoreductive RFA/sunitinib arm at 27.2 months compared to sunitnib only arm at 22.5 months and interferon arm at 19.5 months. OS was significantly longer in the cRFA/sunitinib group compared with the sunitinib-alone group (HR = 0.71; P = 0.04) [39].

### Palliation (Figure 3)

**Fig. 3:**
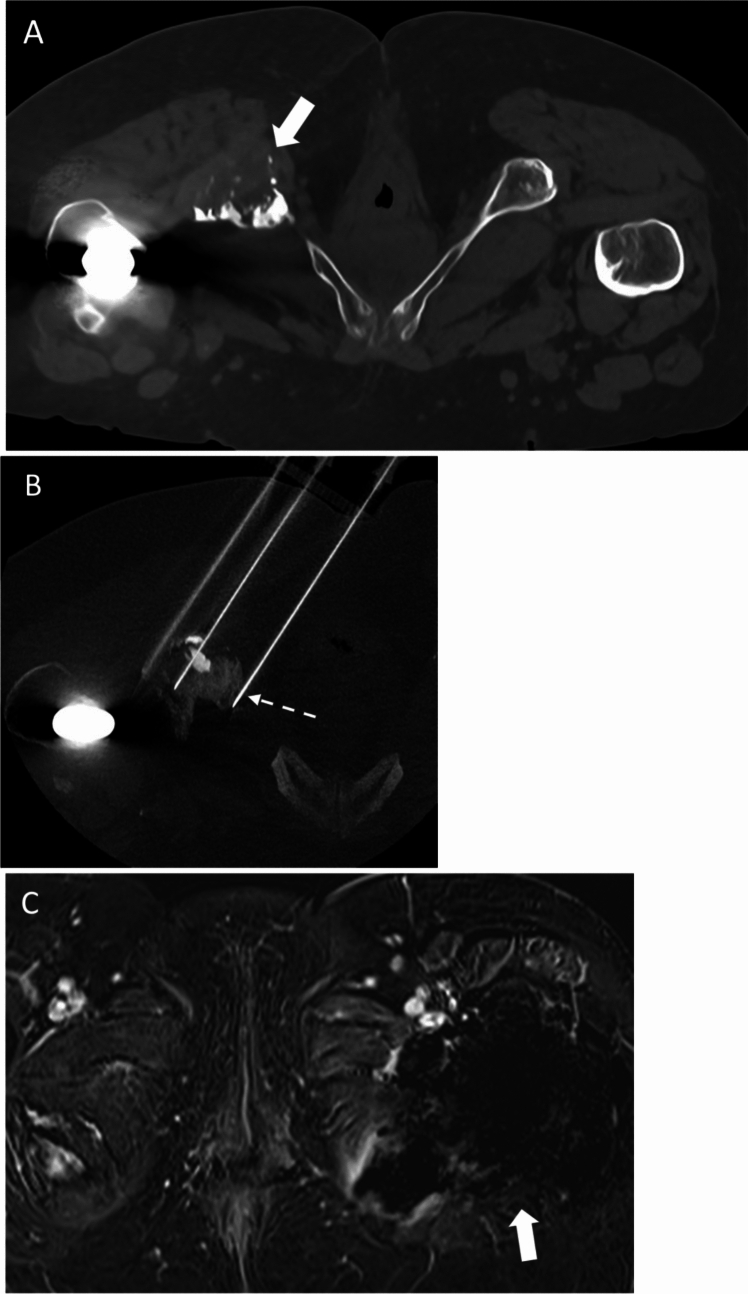
A 56-year-old man with advanced RCC and painful right ischial bone metastasis. **a** Unenhanced axial CT pelvis showed aggressive lytic bone metastasis at right ischium. **b** Under CT guidance, electrochemotherapy, i.e. reversible electroporation with intravenous bleomycin administration, was used to treat the right ischial bone metastasis. Electroporation needle was indicated by *dashed white arrow*. **c** Axial T1-weighed post-contrast MR pelvis showed necrosis of right ischial metastasis. The patient remained opioid-free for 6 months

Osseous metastases are common in metastatic RCC, occurring in about 30 – 40% clear cell RCC patients [40]. Skeletal-related events such as pain refractory to medical therapy and pathological fracture can be debilitating and significantly compromise the patient’s quality of life. Surgical metastasectomy can be very challenging due to the highly vascular nature of these lesions, particularly in the spinal and pelvic locations, with significant morbidity [41]. Radiotherapy is also effective in symptomatic palliation for bone pain; however, the median time to response may take up to three to four weeks [42]. Thermal ablation, including radiofrequency ablation (RFA) and cryoablation, and electrochemotherapy have been shown to be effective in rapid relief of bone tumour pain and improvement of quality of life in several retrospective series (Figure 3) [32, 43–46]. Both RFA and cryoablation demonstrated reduction in pain level in around 64 – 69.7% of patients and significant improvement in quality of life [47]. Bang et al explored the adjunctive cost-effectiveness ratio of multisite cryoablation (MCA) as a palliative measure in mRCC patients. MCA was found to be cost-effective with its cost per life-year gained being USD 44657, compared to sunitinib costing USD 50707. [32]. Although no recent data compared the cost of cryoablation versus ICI-based immunotherapy, given the significantly higher cost of ICI with respective to sunitinib, the cost-effectiveness of cryoablation is expected to be maintained in the current era of ICI-based immunotherapy. An interdisciplinary consensus on the management of bone metastases from renal cell carcinoma published in June 2018 recommended radiofrequency ablation, microwave ablation and cryoablation as treatment options in individual patients with bone metastases <3 cm [48] .

## Conclusion and Future directions

This review summarised the current evidence and roles of image-guided thermal ablation in management of metastatic renal cell carcinoma in the era of immunotherapy-based therapy. Image-guided thermal ablation is feasible and safe and serves as an important adjunct for local disease control in mRCC to improve patient outcome and reduce systemic treatment-related adverse events. However, there is a need of higher-quality prospective data to further evaluate the additional efficacy of image-guided thermal ablation compared to surgical metastasectomy and SBRT, and to identify which patient groups are likely to benefit the most.
